# Women's autonomy in household decision-making and safer sex negotiation in sub-Saharan Africa: An analysis of data from 27 Demographic and Health Surveys

**DOI:** 10.1016/j.ssmph.2021.100773

**Published:** 2021-03-17

**Authors:** Abdul-Aziz Seidu, Richard Gyan Aboagye, Joshua Okyere, Wonder Agbemavi, Mawulorm Akpeke, Eugene Budu, Farrukh Ishaque Saah, Vivian Tackie, Bright Opoku Ahinkorah

**Affiliations:** a Department of Population and Health, University of Cape Coast, Cape Coast, Ghana; b College of Public Health, Medical and Veterinary Services, James Cook University, Australia; c School of Public Health, University of Health and Allied Sciences, Ho, Ghana; d Department of Epidemiology and Biostatistics, School of Public Health, University of Health and Allied Sciences, Hohoe, Ghana; e Department of Nursing, School of Nursing and Midwifery, University of Health and Allied Sciences, Ho, Ghana; f School of Public Health, Faculty of Health, University of Technology Sydney, Sydney, Australia

**Keywords:** Women's autonomy in household decision-making, Married women, Safer sex negotiation, Sub-Saharan Africa, Demographic and health survey, Public health

## Abstract

Women's ability to negotiate the conditions and timing of sex is key to several reproductive health outcomes including family planning and prevention of sexually transmitted infections. We investigated the association between women's autonomy in household decision-making and safer sex negotiation (SSN) in sub-Saharan Africa (SSA). This was a cross-sectional analysis of data from the Demographic and Health Survey (DHS) of 27 countries in SSA. Data were analyzed using Stata version 16.0 using descriptive statistics, chi square test, and logistic regression models. Statistical significance was set at p < 0.05 at 95% confidence interval. The pooled prevalence of SSN in the 27 countries was 77.1%. Compared to women with low autonomy in household decision-making, those with medium (aOR = 1.30; CI = 1.23–1.37) and high levels of autonomy in household decision-making (aOR = 1.28; CI = 1.17–1.40) were more likely to have greater SSN. Those with primary (aOR = 1.35; CI = 1.28–1.41) and secondary/higher education level of education (aOR = 1.68; CI = 1.58–1.79) had higher odds of SSN, compared to those with no formal education. Women who were working had higher odds of SSN (aOR = 1.44; CI = 1.37–1.51) than those who were not working. Women in the middle (aOR = 0.93; CI = 0.87–0.99) and richer (aOR = 0.92; CI = 0.85–0.98) wealth status had lower odds of SSN, compared to those in the poorest wealth status. Women's autonomy in household decision-making is a significant predictor of SSN. Women autonomy in household decision-making programs and interventions should be intensified to achieve Sustainable Development Goals 3.7 and 5 which seek to achieve universal access to sexual and reproductive health services and ensure gender equality and empower all women and girls by 2030.

## Highlights

• Women's ability to negotiate the conditions and timing of sex with their partners is key to control several reproductive health outcomes.
• Women's inability to negotiate for safer sex in many low- and middle-income countries puts them at higher risk of getting infected with STIs including HIV/AIDS.
• In the present study, women with medium and high levels of autonomy in household decision-making were more likely to have greater safer sex negotiation.
• Women who had primary, secondary or higher education were more likely to have safer sex negotiation.
• Women's decision-making autonomy programs and interventions should be intensified to achieve Sustainable Development Goals 3.7.

## Background

Women's ability to negotiate the conditions and timing of sex with their partners is key to the control of a number of reproductive health outcomes (Wolff, Blanc, & Gage, 2000). Ung et al. (2014) reported that women's household decision making in terms of negotiating for safer sex is an important determinant of their vulnerability and resilience to new HIV infections. Tenkorang (2012) also reported that the vulnerability of married women to HIV infection is linked to several factors, including their inability to ask their husbands to use condoms or refusing sexual intercourse even in high-risk situations. HIV/AIDS, other STIs, and unintended pregnancies are major issues of concern in low-and middle-income countries which lead to disability-adjusted life years lost for women of reproductive age (Jesmin & Cready, 2014).

Brunson, Shell-Duncan, and Steele (2009) explained women's household decision-making autonomy, an aspect of women's autonomy, as women's ability to control and make decisions about their bodies and resources without the need for permission within a marital relationship. For example, married women's contribution to their healthcare, movement in terms of visits to family and friends, and capability of negotiating with their partners for safer sex or otherwise are key determinants of their autonomy (Ung et al., 2014).

Women's inability to negotiate for safer sex in many low- and midddle-income countries puts them at higher risk of getting infected with STIs including HIV/AIDS (Feyisetan & Oyediran, 2019; Jesmin & Cready, 2014). The risks to HIV infection are related to sexual attitudes, beliefs, and power dynamics among women and men within marriage. Women's higher risk of being infected with HIV can be associated with cultural beliefss that justify men's action to allow them to enjoy more sexual freedom than women within marriages (Sano, Sedziafa, Vercillo, Antabe, & Luginaah, 2018).

Some married women in sub-Saharan Africa encounter barriers in negotiating for safer sex such as refusing sex and asking for condom use with their partners, even when they perceive partners' risky extra-marital sexual behaviours. For instance, a woman asking her husband to use a condom can be challenging because women often face high expectations of child-bearing (Bauni & Jarabi, 2003; Maharaj & Cleland, 2004). According to Wolff et al. (2000), the acceptance of polygyny and bride wealth in many African communities have the potential to seriously constrain women's ability to negotiate safer sex with their husbands.

Similarly, refusing sex within marriage is often difficult as it can lead to marital dissolution, which is highly stigmatized in most African patriarchal societies (Wolff et al., 2000). Feyisetan and Oyediran (2019) reported that being a married woman in a male-dominated culture like Nigeria and Cote d’Ivoire exacerbates women's risk of STI contraction since society traditionally expects women to be subservient to their husbands, thus exposing them to unprotected sex from husbands who do not believe in protected sex.

Richer and more educated women in urban settings are more likely to have control over their reproductive and sexual choices than their poorer and less educated counterparts who might be living in the rural settings (Amoyaw, Kuuire, Boateng, Asare-Bediako, & Ung, 2015; Tenkorang, 2012). The reason being that those richer and more educated women who are working may not necessarily have to depend on their partners economically. A study conducted in Nigeria reported that due to women's lower socioeconomic status, married women with lower household-decision making autonomy face barriers in negotiating safer sex with partners (Sano et al., 2018). Whereas women's autonomy in household decision-making has gained much attention as a means of enhancing their lives and that of their families (Allendorf, 2012), empirical evidence on women's autonomy in household decision-making and safer sex negotiation (SSN) in sub-Saharan Africa is limited. It is against this backdrop that our study seeks to investigate the association between women's autonomy in household decision-making and safer sex negotiation in SSA.

Our study was guided by the theory of Gender and Power (Connell, 1987) and the Sexual Script Framework (Gagnon & Simon, 2005). The gender and power theory is centered on the idea that sexual practices which encompases safer sex negotiation result from cosequences of unequal power relations that are structurally embedded in a patriarchal system (Feyisetan & Oyediran, 2019). Social practices such as uneven distribution of power in marital relationships, acceptance of male promiscuity, restriction of women's mobility and women's submissiveness to their partners' sexual needs increase their vulnerability to risky sexual behaviours which will require a great deal of women's independence and decision-making power to be able to overcome such predicament (risky sexual behaviours) (Feyisetan & Oyediran, 2019). The sexual script framework on the other hand posits that societies provide cultural scenarios that prescribe appropriate sexual behaviour (Feyisetan & Oyediran, 2019; Gagnon & Simon, 2005). In view of this, sexual negotiation between husbands and wives would be determined by the congruence between one's cultural scenarios, interpersonal scripts and intrapsychic scripts (Feyisetan & Oyediran, 2019). The two frameworks guided our investigation of the effect of women's autonomy in household decision-making on safer sex negotaiation among women in union in sub-Saharan Africa.

## Methods

### Data source and study design

This study involved a cross-sectional analysis of data from the Demographic and Health Survey (DHS) of twenty-seven (27) countries in sub-Saharan Africa. Specifically, the data used was extracted from the women's recode (IR) file which contains data on women from 15 to 49 years. The DHS is a nationally representative survey that is carried out globally in over eighty-five (85) low-and-middle-income countries. The survey collects data on men, maternal, and child health issues (Corsi, Neuman, Finlay, & Subramanian, 2012). A two-stage stratified sampling technique was employed to collect the nationally representative data from the respondents. A detailed explanation of the sampling procedure has been highlighted in a study by Aliaga and Ruilin (2006). In the present study, a total of 133,678 married/cohabiting women aged 15–49 with complete data on the variables of interest were included in the final analysis. A detailed description of the sample extracted for the study can be found in Table 1. The dataset is freely available for download at https://dhsprogram.com/data/available-datasets.cfm. We relied on the “Strengthening the Reporting of Observational Studies in Epidemiology” (STROBE) guideline in writing the manuscript (Knottnerus & Tugwell, 2008).

**Table 1:** Distribution of study sample

Countries	Year of survey	Weighted (N)	Weighted (%)	Can refuse sex	Can ask for condom use	SSN	Autonomy in household decision-making		
							Low	Medium	High
Burkina Faso	2010	9826.0	7.4	55.2	40.4	63.1	63.2	35.5	1.3
Benin	2018	3116.0	2.3	60.4	45.4	64.7	73.0	22.6	4.4
Burundi	2016–17	8538.0	6.4	60.9	60.7	78.2	81.7	13.7	4.6
Congo DR	2013–14	6696.0	5.0	69.7	48.3	78.9	68.9	26.8	4.3
Congo	2013	4107.0	3.1	71.7	70.4	87.2	51.9	44.5	3.6
Cote d’Ivoire	2011–12	2754.0	2.1	63.3	51.3	72.3	73.7	23.2	3.1
Cameroon	2018	5607.0	4.2	70.7	53.4	74.5	79.8	17.3	2.8
Ethiopia	2016	5367.0	4.0	48.4	40.2	57.2	74.9	19.9	5.2
Gabon	2012	2589.0	1.9	85.8	87.1	94.7	36.8	53.5	9.7
Ghana	2014	3614.0	2.7	77.1	72.2	85.8	56.1	36.3	7.6
Gambia	2013	4179.0	3.1	56.9	48.4	65.4	65.1	32.9	2.0
Guinea	2018	2584.0	1.9	53.8	35.7	59.6	72.8	22.0	5.2
Kenya	2014	6800.0	5.1	77.4	79.2	88.5	49.4	43.4	7.2
Comoros	2012	1337.0	1.0	56.2	57.3	68.7	63.1	23.5	13.4
Liberia	2013	3492.0	2.6	86.2	61.0	89.6	66.1	28.0	5.9
Lesotho	2014	1387.0	1.0	76.5	96.1	97.7	42.2	51.6	6.2
Mali	2018	4566.0	3.4	29.5	34.8	45.2	81.9	16.4	1.8
Malawi	2015–16	13,154.0	9.8	70.6	76.0	82.7	71.9	24.2	3.9
Namibia	2013	2235.0	1.7	95.3	97.0	99.0	38.5	50.7	10.8
Rwanda	2014–15	6141.0	4.6	83.8	85.0	93.9	68.9	25.2	5.9
Sierra Leone	2019	5962.0	4.5	71.9	52.3	75.7	79.1	16.8	4.1
Senegal	2010–11	5477.0	4.1	32.7	36.8	47.8	78.4	18.5	3.1
Chad	2014–15	985.0	0.7	66.4	42.8	72.8	62.2	33.9	3.9
Togo	2013–14	3572.0	2.7	77.1	69.6	84.3	72.5	24.2	3.3
Uganda	2016	8619.0	6.5	86.7	81.4	92.3	59.1	33.2	7.7
Zambia	2018	5869.0	4.4	65.3	73.5	79.8	48.7	45.2	6.1
Zimbabwe	2015	5105.0	3.8	72.6	72.7	86.4	47.4	43.0	9.6
**All Countries**		**133,678**	**100.0**	**67.1**	**61.9**	**77.1**	**66.1**	**29.0**	**4.9**

*SSN=Safer Sex Negotiation.

### Variables studied

#### Outcome variable

The main outcome variable was SSN. This variable was assessed as an index from two questions which consisted of “whether married/cohabiting women can refuse sex with their partners” and “whether married/cohabiting women can ask their partners to use condom during sex”. The response options in both questions were 1 = No; 2 = Yes; and 3 = don't know/not sure/depends. For this study, the respondents who responded “Don't know/not sure/depends” were dropped. Therefore, the final response options used in the analysis were 1 = No; and 2 = Yes. A third variable called the SSN was created using the responses from the two questions (can refuse sex and can ask their partner to use condoms). The SSN variable was coded as “1” if the woman could either “refuse sex” or “ask her partner to use condoms” or both and “0” if the woman cannot do any of them. The selection of the variables and their recoding were informed by literature (Putra, Dendup, & Januraga, 2020; Sano et al., 2018; Tenkorang, 2012) and their availability in the datasets.

#### Explanatory variable

Women's autonomy in household decision-making was the main explanatory variable. This was created from three variables measuring women's participation in deciding (1) their health care; (2) household purchases; and (3) visit to family or relatives. All three variables had the same response format. The response options were 1 = respondent alone; 2 = respondent and husband/partner; 3 = husband/partner alone; 4 = someone else; and 5 = other. The responses were further recoded into “yes” for women whose response option was “1” and “no” to those whose response options were “2, 3, 4, and 5”. An index variable was created and we termed it as “women's autonomy in household decision-making”. A composite score was then generated ranging from “0” to “3”. An index score of “0” = no autonomy in household decision-making; “1–2” = medium autonomy in household decision-making; and “3” = high autonomy in household decision-making. The variables used to determine women's autonomy in household decision-making, as well as its scoring, were selected based on previous studies (Atteraya, Kimm, & Song, 2014; Putra et al., 2020).

### Covariates

A total of 14 covariates were selected and included in the study. These variables were selected based on their availability in the dataset and their significant association with SSN from previous studies (Atteraya et al., 2014; Feyisetan & Oyediran, 2019; Putra et al., 2020; Sano et al., 2018; Tenkorang, 2012; Ung et al., 2014). The variables studied consisted of maternal age, husband/partner's age, marital status, maternal educational level, husband/partner's educational level, wealth status, employment status, religion, place of residence, mass media exposure (reading newspaper/magazine, listening to radio, watching television), HIV testing, and comprehensive HIV/AIDS knowledge. This study utilized the already pre-coded responses in the DHS for maternal age, wealth status, employment status, place of residence, wealth status, and HIV testing. The level of education was coded as no education, primary, secondary and higher in the DHS. However, in the present study, maternal and husband/partner's educational level were recoded as no education, primary and secondary/higher. The husband/partner's age was recoded as 15–19; 20–24; 25–29; 30–34; 35–39; 40–44; and 45 years and above. Marital status was coded as married and cohabiting. Religious affiliation was coded as "Christianity, Islam, Traditional, No religion, and other. Each of mass media exposure variables (frequency of reading newspaper/magazine, frequency of listening to radio, and frequency of watching television) was categorized into “not at all, less than once a week and at least once a week”, which were re-categorized into "No" (not at all) and “Yes” (less than once a week and at least once a week). Lastly, comprehensive HIV/AIDS knowledge was coded as “Yes” and “No”.

### Statistical analyses

Data analyses were performed using Stata version 16.0 (Stata Corporation, College Station, TX, USA). The analyses were carried out in four steps. In the first analysis, percentages were used to present the result of SSN and women autonomy in household decision-making as shown in Table 1. Secondly, a bivariate analysis using chi-square test of independence was performed to determine the proportions of SSN practices across women autonomy in household decision-making and covariates (Table 2). In the third phase of the analysis, bivariate and multivariable logistic regression were carried out to determine the association between SSN and women autonomy in household decision-making, adjusted for all the covariates. Similarly, the last analysis was performed to determine the effect of women autonomy in household decision-making on SSN in all the 27 countries through bivariate and multivariable logistic regression analysis (Table 3). The results of the regression analyses were presented using crude odds ratios (cOR) and adjusted odds ratios (aOR) and their respective 95% confidence intervals (CIs). Statistical significance was set at p < 0.05. A multicollinearity test was conducted using the variance inflation factor (VIF). A mean VIF of 2.40 was found, showing no evidence of multicollinearity among the variables studied. The women's sample weights (v005/1,000,000) were applied to obtain unbiased estimates according to the DHS guidelines and the survey command (svy) in Stata was used to adjust for the complex sampling structure of the data in both the chi-square and regression analyses.

**Table 2:** Background characteristics, autonomy in household decision-making, and safer sex negotiation among women in SSA

Variable	Weighted N	Weighted %	Can refuse sex		Can ask for condom use		SSN	
			Yes^a^	p-value	Yes^a^	p-value	Yes^a^	p-value
**Autonomy in household decision-making**				<0.001		<0.001		<0.001
Low	88,286	66.1	64.5		58.9		74.3	
Medium	38,795	29.0	71.9		67.3		82.4	
High	6597	4.9	74.0		70.2		83.4	
**Maternal age**				<0.001		<0.001		<0.001
15–19	7855	5.9	61.8		58.3		72.1	
20–24	23,904	17.9	67.7		64.4		78.4	
25–29	30,009	22.5	68.2		64.2		78.6	
30–34	25,999	19.4	68.1		63.3		78.3	
35–39	21,312	15.9	67.4		61.1		76.8	
40–44	14,405	10.8	66.0		58.9		75.5	
45–49	10,194	7.6	64.8		54.3		73.2	
**Partner's age**				<0.001		<0.001		<0.001
15–19	520	0.4	65.1		64.1		75.7	
20–24	7521	5.6	70.3		68.9		81.1	
25–29	19,078	14.3	70.2		67.5		80.9	
30–34	24,768	18.5	69.3		66.6		80.3	
35–39	23,878	17.9	68.4		64.2		78.6	
40–44	20,215	15.1	67.4		61.7		77.4	
45+	37,698	28.2	62.5		53.1		71.2	
**Marital status**				<0.001		<0.001		<0.001
Married	107,212	80.2	64.3		58.8		74.5	
Cohabiting	26,466	19.8	78.6		74.2		87.7	
**Maternal educational level**				<0.001		<0.001		<0.001
No education	43,888	32.8	53.8		41.0		61.8	
Primary	47,068	35.2	70.1		67.9		81.5	
Secondary or higher	42,722	32.0	77.5		76.7		87.9	
**Partner's educational level**				<0.001		<0.001		<0.001
No education	37,744	28.2	52.1		40.3		60.4	
Primary	40,664	30.4	69.8		67.0		80.8	
Secondary or higher	55,270	41.4	75.3		72.8		85.8	
**Wealth status**				<0.001		<0.001		<0.001
Poorest	23,093	17.3	62.7		52.2		71.7	
Poorer	25,786	19.3	64.4		56.5		73.5	
Middle	26,392	19.7	65.2		59.9		75.1	
Richer	27,992	20.9	67.3		64.4		78.5	
Richest	30,415	22.8	74.1		73.2		84.8	
**Employment status**				<0.001		<0.001		<0.001
Not working	34,679	25.9	61.8		57.6		71.4	
Working	98,999	74.1	69		63.4		79.1	
**Religion**				<0.001		<0.001		<0.001
Christianity	89,205	66.7	73.8		70.6		84.3	
Islam	39,663	29.7	52.1		43.6		61.2	
Traditionalist	1995	1.5	62.8		36.5		68.9	
No religion	2024	1.5	65.2		54.9		74.5	
Other religion	791	0.6	77.9		75.4		85.6	
**Comprehensive HIV/AIDS knowledge**				<0.001		<0.001		<0.001
No	73,886	55.3	62.2		55.2		72.3	
Yes	59,792	44.7	73.1		70.2		83.1	
**HIV testing**				<0.001		<0.001		<0.001
No	40,258	30.1	54.9		41.7		63.2	
Yes	93,420	69.9	72.4		70.6		83.1	
**Reads newspaper/magazine**				<0.001		<0.001		<0.001
No	107,954	80.8	64.5		57.5		74.1	
Yes	25,724	19.2	78.2		80.1		89.5	
**Listens to radio**				<0.001		<0.001		<0.001
No	49,664	37.2	63.3		55.5		72.8	
Yes	84,014	62.8	69.4		65.7		79.6	
**Watches television**				<0.001		<0.001		<0.001
No	78,821	59.0	65.4		57.4		74.7	
Yes	54,857	41.0	69.6		68.3		80.5	
**Place of residence**				<0.001		<0.001		<0.001
Urban	47,509	35.5	72.8		69.6		83.1	
Rural	86,169	64.5	64.0		57.6		73.8	

Note.

a **=** Reported row percentages.

**Table 3:** Autonomy in household decision-making and safer sex negotiation among women in SSA

Variables	Model 1	Model II
	cOR [95%CI]	aOR [95%CI]
**Autonomy in household decision-making**		
Low	[1.00,1.00]	[1.00,1.00]
Medium	1.61*** [1.53–1.70]	1.30*** [1.23–1.37]
High	1.73*** [1.58–1.89]	1.28*** [1.17–1.40]
**Maternal age**		
15–19	[1.00,1.00]	[1.00,1.00]
20–24	1.40*** [1.31–1.50]	1.18*** [1.09–1.28]
25–29	1.43*** [1.33–1.53]	1.26*** [1.15–1.37]
30–34	1.40*** [1.30–1.50]	1.31*** [1.21–1.45]
35–39	1.28*** [1.19–1.37]	1.36*** [1.23–1.51]
40–44	1.20*** [1.11–1.29]	1.38*** [1.24–1.54]
45–49	1.06 [0.98–1.15]	1.38*** [1.24–1.55]
**Partner's age**		
15–19	[1.00,1.00]	[1.00,1.00]
20–24	1.38 [1.00–1.89]	1.09 [0.78–1.49]
25–29	1.36 [0.99–1.85]	0.99 [0.72–1.36]
30–34	1.31 [0.96–1.79]	0.94 [0.68–1.29]
35–39	1.18 [0.86–1.61]	0.87 [0.63–1.19]
40–44	1.10 [0.80–1.50]	0.83 [0.60–1.14]
45+	0.79 [0.58–1.08]	0.73 [0.53–1.12]
**Marital status**		
Married	[1.00,1.00]	[1.00,1.00]
Cohabiting	2.45*** [2.28–2.64]	1.61*** [1.50–1.73]
**Maternal educational level**		
No education	[1.00,1.00]	[1.00,1.00]
Primary	2.72*** [2.59–2.85]	1.35*** [1.28–1.41]
Secondary/higher	4.50*** [4.24–4.77]	1.68*** [1.58–1.79]
**Partner's educational level**		
No education	[1.00,1.00]	[1.00,1.00]
Primary	2.75*** [2.61–2.90]	1.31*** [1.24–1038]
Secondary/higher	3.96*** [3.74–4.19]	1.49*** [1.41–1.57]
**Wealth status**		
Poorest	[1.00,1.00]	[1.00,1.00]
Poorer	1.09** [1.04–1.15]	0.96 [0.91–1.01]
Middle	1.19*** [1.12–1.26]	0.93* [0.87–0.99]
Richer	1.44*** [1.34–1.53]	0.92* [0.85–0.98]
Richest	2.19*** [2.03–2.37]	0.97 [0.88–1.06]
**Employment status**		
Not working	[1.00,1.00]	[1.00,1.00]
Working	1.51*** [1.44–1.59]	1.44*** [1.37–1.51]
**Religion**		
Christianity	[1.00,1.00]	[1.00,1.00]
Islam	0.29**** [0.28–0.31]	0.57*** [0.53–0.60]
Traditionalist	0.41*** [0.35–0.48]	0.92 [0.79–1.08]
No religion	0.54*** [0.47–0.63]	0.84* [0.73–0.97]
Other religion	1.10 [0.84–1.45]	0.99 [0.75–1.30]
**Comprehensive HIV/AIDS knowledge**		
No	[1.00,1.00]	[1.00,1.00]
Yes	1.89*** [1.80–1.97]	1.36*** [1.30–1.43]
**HIV testing**		
No	[1.00,1.00]	[1.00,1.00]
Yes	2.87*** [2.73–3.01]	1.62*** [1.54–1.70]
**Reads newspaper/magazine**		
No	[1.00,1.00]	[1.00,1.00]
Yes	2.98*** [2.79–3.19]	1.32*** [1.23–1.42]
**Listensto radio**	1	1
No	[1.00,1.00]	[1.00,1.00]
Yes	1.46*** [1.40–1.52]	1.26*** [1.20–1.31]
**Watchestelevision**	1	1
No	[1.00,1.00]	[1.00,1.00]
Yes	1.40*** [1.33–1.48]	0.99 [0.94–1.05]
**Place ofresidence**	1	1
Urban	[1.00,1.00]	[1.00,1.00]
Rural	0.58*** [0.54–0.61]	0.84*** [0.79–0.91]

*p < 0.05, **p < 0.01, ***p < 0.001, cOR = Crude Odds Ratio; aOR = Adjusted Odds Ratio; CI=Confidence Interval; [1.00,1.00] = reference category.

### Ethical approval

From the DHS reports, ethical clearances were obtained from the Ethics Committee of ORC Macro Inc. as well as Ethics Boards of partner organizations of the various countries such as the Ministries of Health. The survey was conducted with adherence to the standards for ensuring the protection of respondents' privacy. Inner City Fund International ensures that the survey complies with the U.S. Department of Health and Human Services’ regulations for the respect of human subjects. This was a secondary analysis of data and therefore no further approval was required since the data is available in the public domain. Further information about the DHS data usage and ethical standards are available at http://goo.gl/ny8T6X.

## Results

The pooled prevalence of SSN in the 27 countries was 77.1%. The bivariate (chi-square) results show that autonomy in household decision-making, as well as all the covariates were all statistically significant in relation to women being able to refuse sex, ask for condom use, and SSN (see Table 2). The prevalence of SSN was highest among women with high autonomy in household decision-making levels (83.4%), those aged 25–29 (78.6%), those whose partners were between ages 20–24 (81.1%), cohabiting women (87.7%), those with secondary/higher education (87.9%), those whose partners also had secondary/higher education (85.8%), women in the richest wealth status (84.8%), those working (79.1%), women who professed other religions (85.6%), those who were resident in urban areas (83.1%), those who read magazine/newspapers (89.5%), listened to radio (79.6%), and watched television (80.5%).

### Multivariable logistic regression analysis on autonomy in household decision-making and safer sex negotiation among women in sub-Saharan Africa

Findings from the logistic regression analysis of the association between women's autonomy in household decision-making and SSN are presented in Table 3. The results show that after controlling for the covariates, women with medium [aOR = 1.30; CI = 1.23–1.37] and high levels of autonomy in household decision-making [aOR = 1.28; CI = 1.17–1.40] were more likely to have greater SSN, compared to those with low level of autonomy in household decision-making. Concerning maternal age, it can be seen from the findings that women in all the age categories had higher odds of SSN, compared to those within the age group 15–19 years. Co-habiting women [aOR = 1.61; CI = 1.50–1.73] had a higher likelihood of SSN, compared to married women. Likewise, those with primary [aOR = 1.35; CI = 1.28–1.41] and secondary/higher education [aOR = 1.68; CI = 1.58–1.79] had higher odds of SSN, compared to those with no formal education. The findings also indicate that women whose partners had primary [aOR = 1.31; CI = 1.24–1038] and secondary/higher education [aOR = 1.49; CI = 1.41–1.57] had higher odds of SSN, compared to those with no formal education. Again, compared to women who were not working, women who were working had higher odds of SSN [aOR = 1.44; CI = 1.37–1.51]. Comparatively, women who professed Islam [aOR = 0.57; CI = 0.53–0.60] or No Religion [aOR = 0.84; CI = 0.73–0.97] had lower likelihood of SSN compared to those who professed Christianity. Further, women who had comprehensive knowledge on HIV/AIDS [aOR = 1.36; CI = 1.30–1.43] had higher odds SSN, compared to those with no comprehensive knowledge. Respondents who had tested for HIV [aOR = 1.62; CI = 1.54–1.70] had higher likelihood of SSN, compared to those who have not tested for HIV. Also, respondents in the middle [aOR = 0.93; CI = [0.87–0.99] and richer [aOR = 0.92; CI = 0.85–0.98] wealth status had lower odds of SSN, compared to those in the poorest wealth status. Women who read newspaper/magazine [aOR = 1.32; CI = 1.23–1.42] and those who listened to radio [aOR = 1.26; CI = 1.20–1.31] had higher odds of SSN, compared to those who neither read newspapers/magazine nor listen to radio.

### Logistic regression on the association between women autonomy in household decision-making and safer sex negotiation among women in sub-Saharan Africa by countries

Table 4 also shows the findings of the logistic regression on the association between women autonomy in household decision-making and SSN among women in sub-Saharan Africa. Results from the analysis shows that women with medium/high autonomy in household decision-making were more likely to negotiate for safer sex in Cameroon, Congo, Congo DR, Benin, Cote D'lvoire, Ghana, Guinea, Liberia, Senegal, Sierra Leone, Mali, Ethiopia, Kenya, Uganda, Zambia and Malawi. However, the odds of having SSN was less likely among women in Burkina Faso [aOR = 0.90; CI = 0.82–0.99], the Gambia [aOR = 0.83; CI = 0.72–0.95], and Burundi [aOR = 0.87; CI = 0.76–0.99] had lower odds of SSN.

**Table 4:** Logistic regression on the association between women autonomy in household decision-making and safe sex negotiation among women in sub-Saharan Africa by countries

Countries	Model I	Model II
	cOR [95%CI]	aOR [95%CI]
**Central Africa**		
Cameroon	1.99*** [1.67–2.37]	1.38** [1.14–1.67]
Chad	0.99 [0.73–1.34]	0.71 [0.49–1.01]
Congo	1.38** [1.14–1.67]	1.35** [1.11–1.65]
Congo DR	1.42*** [1.24–1.63]	1.29*** [1.12–1.49]
Gabon	1.23 [0.90–1.68]	0.88 [0.63–1.23]
**West Africa**		
Benin	1.86*** [1.56–2.22]	2.16*** [1.79–2.61]
Burkina Faso	1.04 [0.96–1.14]	0.90* [0.82–0.99]
Cote D'lvoire	1.74*** [1.41–2.15]	1.33* [1.05–1.68]
Gambia	0.95 [0.83–1.08]	0.83** [0.72–0.95]
Ghana	1.26* [1.04–1.53]	1.34** [1.09–1.64]
Guinea	1.59*** [1.33–1.91]	1.46*** [1.21–1.78]
Liberia	1.83*** [1.43–2.35]	1.49** [1.15–1.94]
Mali	1.60*** [1.36–1.89]	1.47*** [1.24–1.74]
Sierra Leone	1.49*** [1.27–1.74]	1.49*** [1.27–1.76]
Senegal	1.59*** [1.38–1.83]	1.35*** [1.16–1.57]
Togo	1.02 [0.85–1.22]	1.00 [0.82–1.22]
**East Africa**		
Burundi	0.85* [0.74–0.96]	0.87* [0.76–0.99]
Comoros	1.08 [0.86–1.35]	0.96 [0.75–1.22]
Ethiopia	1.56*** [1.37–1.78]	1.30*** [1.22–1.49]
Kenya	1.73*** [1.51–1.99]	1.54*** [1.32–1.80]
Rwanda	0.81 [0.65–1.00]	0.81 [0.65–1.01]
Uganda	1.29** [1.10–1.51]	1.31** [1.11–1.54]
Zambia	1.42*** [1.25–1.61]	1.34*** [1.18–1.53]
**Southern Africa**		
Lesotho	1.13 [0.56–2.29]	0.93 [0.44–1.98]
Malawi	1.17** [1.05–1.30]	1.15* [1.03–1.28]
Namibia	1.78 [0.85–3.71]	1.23 [0.53–2.85]
Zimbabwe	1.09 [0.93–1.29]	1.07 [0.91–1.27]

Model1: Unadjusted model examining the independent association of women autonomy in household decision-making and safe sex negotiation.

Model II: Adjusted for maternal age, paternal age, marital status, maternal education, paternal education, wealth, employment, religion, comprehensive HIV/AIDS knowledge, HIV testing, newspaper, radio, television, and residence.

*p < 0.05, **p < 0.01, ***p < 0.001, cOR = Crude Odds Ratio; aOR = Adjusted Odds Ratio; CI=Confidence Interval.

## Discussion

Studies have shown that women's capacity to negotiate for safer sex is quintessential towards the reduction in the prevalence of STIs, including HIV among women (Feyisetan & Oyediran, 2019; Jesmin & Cready, 2014). Yet, research on SSN in sub-Saharan Africa remains sparse. Therefore, we sought to investigate the association between women's autonomy in household decision-making and SSN among women in sub-Saharan Africa using data from the DHS of 27 countries in sub-Saharan Africa. The results indicate that women's autonomy in household decision-making increases the likelihood of being able to negotiate for safer sex in sub-Saharan Africa. Women who had medium or high levels of autonomy in household decision-making were 1.30 and 1.28 times respectively, more likely to have SSN, compared to those with low autonomy in household decision-making. Our finding corroborates with results from previous studies, where women with medium to high autonomy in household decision-making were more likely to have SSN (Putra et al., 2020; Sano et al., 2018; Doku & Asante, 2015; Atteraya et al., 2014; Jennings et al., 2014). A possible explanation for this finding could be that women who are empowered are most likely to engage in household decision-making as well as being involved in decisions that has to do with their health. Hence, such women can insist on safe sex practices such as refusing to have unprotected sex (Boateng et al., 2014). This implies that empowering women is a strategic approach to honing their potential to negotiate for safer sex.

Congruent with previous studies on SSN among women (Acharya, Bell, Simkhada, Van Teijlingen, & Regmi, 2010; Atteraya et al., 2014; Pradnyani & Arief Wibowo, 2019; Putra et al., 2020), our findings indicate that education is a significant covariate of SSN among women, with those with formal education being most likely to negotiate for safer sex. The observed association between education and SSN could be inferred from the assertion that formal education increases the autonomy of women as well as enhance their capacity to participate in decision-making concerning their reproductive health and health in general (Acharya et al., 2010). Another plausible justification for this finding could be that formal education provides women with accurate information concerning STIs and HIV/AIDS and this probably increases women's attitude to practice SSN (Putra et al., 2020). Similar trend of association was found among women whose partners were educated. This could be explained from the point that when partners of women are educated, they become aware of the need for safe sex, as well as respect the autonomy of women to insist on safe sex.

We also found that women who had comprehensive knowledge of HIV were 1.36 times more likely to negotiate for safe sex. This finding is consistent with a related study by Putra et al. (2020). Some studies have shown that women who lack comprehensive knowledge on HIV or perceive HIV to be a myth were more probable to have negative attitudes towards contraceptive use, and are less likely to negotiate for safer sex (Ung et al., 2014). However, when women have comprehensive knowledge on HIV, they appreciate the significance of SSN much better as their perceived risk of HIV becomes heightened. Therefore, they are more likely to negotiate for safer sex. In the same vein, women who have tested for HIV are more likely to negotiate for safer sex. This may be attributed to counseling that they receive prior to the HIV testing. During these counseling sessions, women are educated about the need to practice safe sex as a means of reducing the chances of contracting HIV and other STIs.

Our findings also reveal that cohabiting women, compared to married women, had a higher likelihood of negotiating for safer sex. This finding is supported by previous studies that reported that married women scored less on the probability to negotiate for safe sex (De Coninck, Feyissa, Ekström, & Marrone, 2014). This could probably be explained from the perspective that married women feel obligated to consent to sex from their partners at any point in time, even if it is unprotected. Traditionally, in the African context, females are required by societal expectations and norms to be submissive, particularly when they are married. Therefore, married women are less likely to negotiate for safe sex with their partners, compared to those who are cohabiting, and this exacerbates their risk of STIs and HIV (Tenkorang, 2012).

Moreover, we found from our study that women who frequently read the newspaper/magazine or listened to the radio were more likely to negotiate for safer sex in sub-Saharan Africa. We are not surprised by this finding because through the media like newspaper, magazine, radio and television, women receive information pertaining to STIs, HIV and safe sex practices which informs their decision and increase the odds to negotiate for safer sex. Our findinding could be inferred from the assertion that women become enlightened and empowered to refuse sex or insist on condom use during sex when exposed to adequate information from the media (Ampofo, 2001).

It is also clear from our findings that women who were working had higher chances of negotiating safe sex, compared to their counterparts who were not working. The result is consistent with the findings of De Coninck et al. (2014) who revealed that women who were employed between 10 and 30 percent times more likely to report SSN. This could be justified by the financial and psychological independence that employed women experience, thereby increasing their potential to negotiate for safer sex. This result also corroborates our findings that women in the middle and richer wealth status have a higher likelihood of negotiating for safe sex. As women become employed, their economic/wealth status increases and that empowers them to take critical decisions about their health, including SSN (Ung et al., 2014; Tenkorang, 2012).

We also observed from our study that women who professed Islam or no religion had lower odds of SSN. This implies that Christians are more likely to negotiate for safer sex. The result is supported by Jesmin and Cready (2014) who found from their study that Muslims compared to non-Muslim women were less likely to negotiate for safer sex. This is probably because, Muslim women by virtue of the traditional and ideological perspectives may be less mobile and less vocal about their rights and participation in decision making (Naved & Persson, 2008). Consequently, it reduces their likelihood to negotiate for safer sex.

### Strength and limitation

The major strength of this study lies in the use of a nationally representative dataset, the DHS, for the analysis. This dataset has been used and validated in several studies thereby making the findings of this study very valid and generalizable to women in sub-Saharan Africa. Moreover, the focus on 27 countries in sub-Saharan Africa helps to better understand the nuances between countries with respect to SSN among women. We acknowledge that this study was not without limitations, and as such, interpretations and inferences made from the findings must take cognizant of these limitations. Since the dataset used in the present study adopted a cross-sectional design, causality cannot be deduced or established. Also, the variable for SSN was self-reported, and is thus, prone to social desirability bias.

## Conclusion

We found women autonomy in household decision-making to be a significant predictor of SSN. The findings underscore the need to augment women autonomy in household decision-making programs and interventions in order to promote greater SSN among women in sub-Saharan Africa with special focus on improving education, socioeconomic conditions, and equitable household power relations of women. Also, African countries should strengthen women autonomy in household decision-making intervention by prioritizing those aged between 15 and 19 years, women who are unemployed, and those with no formal education, as they are the most disadvantaged when it comes to negotiating for safer sex. We believe that when autonomy in household decision-making of women is further strengthened in sub-Saharan Africa, and priorities given to the most at-risk groups, then the Sustainable Development Goals, particularly, SDG 3.7 (universal access to sexual and reproductive health [SRH] services), and SDG 5 (achieve gender equality and empower all women and girls) would be achieved.

## Ethical statement

From the DHS reports, ethical clearances were obtained from the Ethics Committee of ORC Macro Inc. as well as Ethics Boards of partner organizations of the various countries such as the Ministries of Health. The survey was conducted with adherence to the standards for ensuring the protection of respondents' privacy. ICF International ensures that the survey complies with the U.S. Department of Health and Human Services’ regulations for the respect of human subjects. This was a secondary analysis of data and therefore no further approval was required since the data is available in the public domain. Further information about the DHS data usage and ethical standards are available at http://goo.gl/ny8T6X.

## Author statement

Conceptualization: AS; RGA; MA; VT; BOA Methodology: AS; RAG; EB; FIS; BOA Software: AS; BOAData curation: RGA; EB; WA; BOA Formal analysis: AS; RGA; EB; BOAWriting- Original draft preparation: AS; RGA; JO; WA; MA; EB; FIS; VT; BOA Validation: AS; WA; MA; EB; FIS; VT; BOA Writing- Reviewing and Editing: AS; RGA; JO; WA; MA; EB; FIS; VT; BOA

## Declarations of interest

None.

## Financial disclosure

There is no funding for this study.
